# Etiology and Clinical Characteristics of Conductive Hearing Loss at a Tertiary Hospital in Sudan

**DOI:** 10.7759/cureus.112896

**Published:** 2026-07-18

**Authors:** Danya Elkanzi, Rayan Mahmoud

**Affiliations:** 1 Faculty of Medicine, University of Medical Sciences and Technology, Khartoum, SDN

**Keywords:** conductive hearing loss, ent, hearing impairment, khartoum, khartoum state, otitis media, otolaryngology, sudan

## Abstract

Background

Conductive hearing loss (CHL) is a significant contributor to hearing impairment, especially in low-income settings. Chronic suppurative otitis media (CSOM) is often a dominant cause in developing regions. This study examined the causes and clinical profiles of patients with CHL at Khartoum Ear, Nose and Throat (ENT) Hospital.

Methods

We conducted a retrospective descriptive cross-sectional study of 106 patients diagnosed with CHL (confirmed by audiometry or tuning-fork testing) who presented between October 2021 and March 2022. Demographic data, etiologies, and associated symptoms were extracted from medical records and analysed descriptively.

Results

The cohort (mean age 27 years) comprised 61 (57.5%) female patients, predominantly young adults with 54 (51%) patients aged 18-35 years. CSOM was the leading cause of CHL, affecting 50 (47.2%) patients, far exceeding other etiologies. Smaller proportions were due to ear canal foreign bodies in seven (6.6%) participants, external auditory polyps in seven (6.6%), otosclerosis in five (4.7%), and various others (each <4%). The majority of cases were acquired, seen in 99 cases (93.4%) and only four (3.8%) were congenital cases. Most patients had mild-to-moderate hearing loss, with 50 (47.2%) patients reporting progressive loss. Common accompanying symptoms included ear discharge in 70 people (66.0%), tinnitus in 62 (58.9%), ear pain in 61 (57.5%), vertigo in 36 (34.0%), and ear itch in 28 (26.4%) participants.

Conclusion

CSOM predominated as the etiology of CHL in this Sudanese ENT hospital, reflecting a high burden of preventable ear infection. The findings underscore the need for early detection and management of CSOM and related conditions to reduce CHL. The data from this study highlights the need for public health intervention in tackling preventable infectious diseases in Khartoum.

## Introduction

Hearing loss is a major global public health concern. As per the World Health Organization (WHO), hearing loss affects hundreds of millions of people worldwide, with a disproportionate burden in low- and middle-income countries [1,2]. Hearing loss can be classified as either conductive or sensorineural in nature. Sensorineural hearing loss (SNHL) describes hearing loss secondary to pathology of the cochlea, auditory nerve, or central nervous system [3]. While conductive hearing loss (CHL) results from issues preventing sound transfer from the outer to the inner ear, including blockages, middle ear fluid, or infections like otitis media, eardrum perforations, growths such as cholesteatoma, otosclerosis, ossicular discontinuity from head trauma, or congenital conditions like aural atresia [4]. These problems can affect the pinna, ear canal, eardrum, or the ossicles of the middle ear [4]. Worldwide, the majority of hearing loss is sensorineural in nature [3], however CHL does account for a significant proportion of hearing loss in the paediatric population [4]. 

Chronic suppurative otitis media (CSOM) is one of the most common causes of CHL in developing countries and is closely associated with poverty, overcrowding, poor hygiene, and limited access to healthcare [2,5]. CSOM is characterised by chronic middle ear inflammation with tympanic membrane perforation and persistent otorrhoea, often leading to progressive hearing loss and serious complications if untreated [6]. According to the WHO, the percentage of CHL due to CSOM can range from 3-80% depending on the country with high prevalence seen in developing nations. As such, WHO has recognised CSOM as a significant contributor to avoidable hearing disability worldwide [7]. 

In Sudan, there is a lack of comprehensive national data regarding the prevalence and causes of CHL. Previous hospital-based studies in Sudan, have suggested that infectious ear diseases, like CSOM, remain a leading cause of hearing impairment, particularly among children and young adults [8,9]. However, in these studies [8,9], data encompassing all age groups and detailing the clinical characteristics of CHL are limited. For example, one of the studies [9] only focuses on the paediatric population in Khartoum and the other study in Rabak City, Sudan [8] does not describe any clinical characteristics that patients presented with. Understanding the local etiological patterns of CHL is crucial for guiding preventive strategies, improving early diagnosis, and optimising management. 

This study aimed to evaluate the etiologies and clinical characteristics of CHL among patients presenting to Khartoum Ear, Nose and Throat (ENT) Hospital over a six-month period. 

## Materials and methods

Research question

What are the etiologies and clinical characteristics of CHL among patients presenting to Khartoum ENT Hospital over six months?

Objectives

The objectives of this study were to identify the most common etiologies of CHL among patients presenting to Khartoum ENT Hospital from October 2021 to March 2022. Additionally, the study aimed to determine the most frequent clinical symptoms and evaluate the predominant severity of hearing loss within this patient cohort during the specified period.

Study design and setting

A retrospective descriptive cross-sectional study was conducted at Khartoum ENT Hospital, Sudan. Medical records of patients presenting between October 2021 and March 2022 were reviewed.

Study population

All patients of any age and sex who were diagnosed with CHL during the study period were included. The inclusion criteria were CHL diagnosed via audiological evaluation using pure-tone audiometry. In those without a pure-tone audiometry reading, we included them if their tuning fork test (Rinne & Weber) indicated a CHL.

Exclusion criteria for this study were those diagnosed with sensorineural or mixed hearing loss as indicated by pure-tone audiometry or tuning fork tests. 

Data collection

Data was extracted from patient medical records using a structured data collection form (appendix). Collected variables included demographic characteristics (age, sex, and residence), etiology of conductive hearing loss, duration and progression of hearing loss, severity of hearing impairment, and associated otological symptoms such as ear discharge, tinnitus, ear pain, vertigo, and ear itching.

Hearing loss severity was classified as mild (26-40 dB), moderate (41-60 dB), severe (61-80 dB), or profound (>80 dB) according to pure-tone audiometry thresholds, in accordance with the WHO 1997 classification. The duration of symptoms was categorised as less than three months or more than three months.

Data analysis

Data were entered and analysed using the IBM SPSS Statistics for Windows, Version 25 (Released 2017; IBM Corp., Armonk, New York, United States). Descriptive statistics were used to summarise the data. Categorical variables were presented as frequencies and percentages, while continuous variables were summarized using ranges.

Ethical considerations

Ethical approval was obtained from the institutional review board of the University of Medical Science and Technology, as well as approval from the Ministry of Health in Khartoum, Sudan, and Khartoum ENT Hospital, to access patient records. The patients’ confidentiality was respected, with no identifiable characteristics being reported. Their data will not be used for anything besides this research.

## Results

Demographic characteristics

A total of 106 patients diagnosed with CHL were included in the study. The mean age of patients was approximately 27 years, with ages ranging from early childhood to elderly adults. The majority of patients were young adults aged between 18 and 35 years. Female patients constituted 57% of the study population.

Residents of Khartoum state constituted 71.2% of the study population, reflecting the hospital’s role as a major referral centre.

Etiology of CHL

The distribution of etiologies of CHL is summarised in Table 1.

**Table 1:** The distribution of etiology in patients with conductive hearing loss

Etiology	Frequency, n (%)
Ca Mastoid bone	2 (1.9%)
Chronic Suppurative Otitis Media (CSOM)	50 (47.2%)
Ear Canal Atresia	4 (3.8%)
Ear Trauma	4 (3.8%)
External Ear Mass	2 (1.9%)
Foreign Body Ear	7 (6.6%)
Glomus Tumour	1 (0.9%)
Infected Preauricular Sinus	1 (0.9%)
Malignant Otitis Externa	1 (0.9%)
Osteoma	3 (2.8%)
Otitis Externa	3 (2.8%)
Otitis Media	4 (3.8%)
Otitis Media with Effusion (OME)	4 (3.8%)
Otosclerosis	5 (4.7%)
Polyp	7 (6.6%)
Pyogenic Granuloma	1 (0.9%)
Rhabdomyosarcoma	1 (0.9%)
Temporal Bone Mass	1 (0.9%)
Tympanosclerosis	1 (0.9%)
Unknown	4 (3.8%)
Total	106 (100.0%)

CSOM was the most common cause (47.2%), accounting for nearly half of all cases. Other identified etiologies included foreign bodies of the ear (6.6%), external auditory canal polyps (6.6%), otosclerosis (4.7%), ear trauma (3.8%), otitis media with effusion (3.8%), and congenital ear canal atresia (3.8%). Neoplastic and inflammatory external ear conditions were observed less frequently.

Regarding progression of hearing loss, 47.2% of participants reported progressive loss of hearing over time, 35.8% reported their conditions as being stationary while the remainder reported intermittent loss of hearing. 

**Figure 1:**
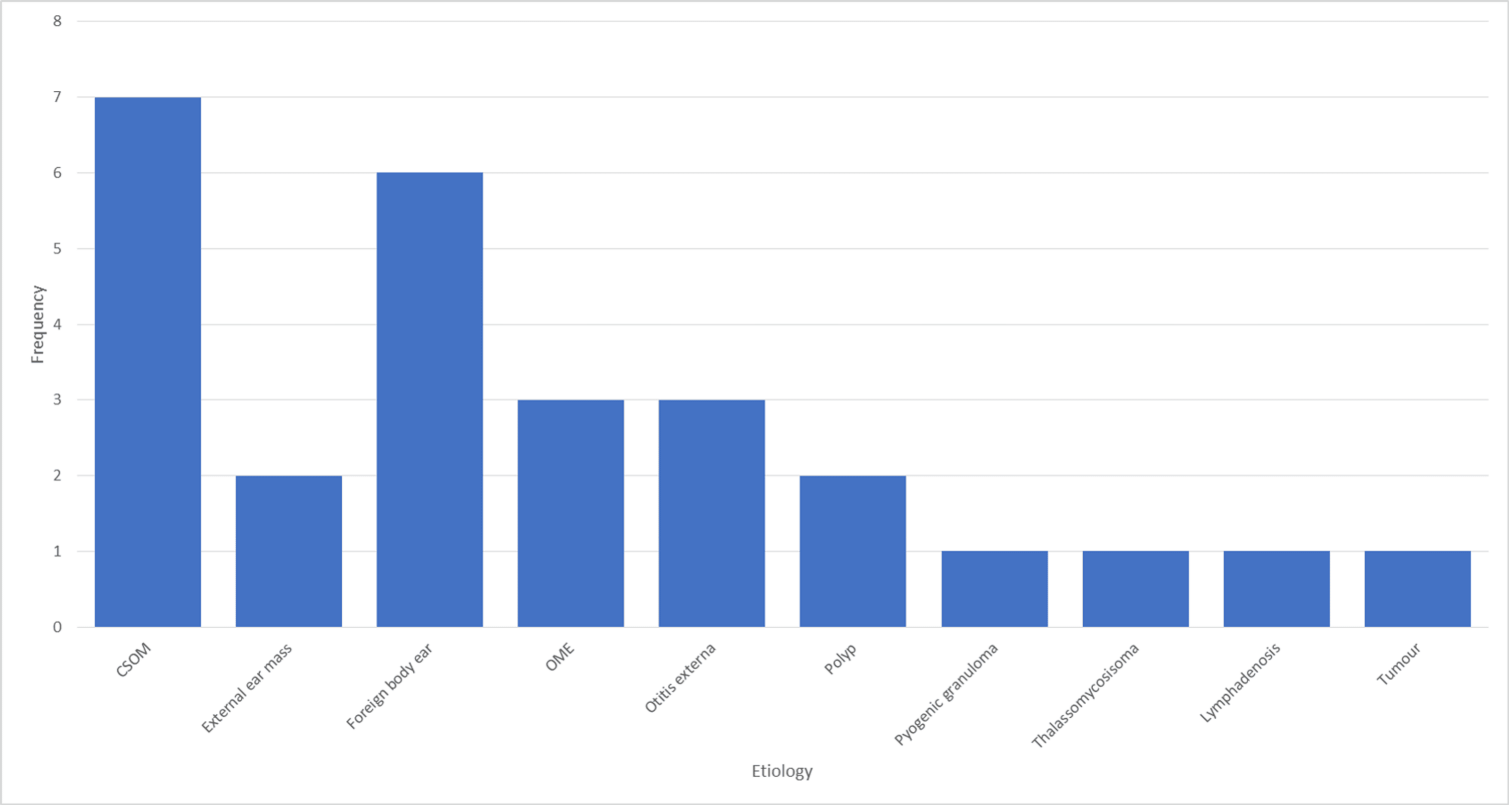
The distribution of etiologies in the paediatric population of the study

Figure 1 represents the distribution of etiologies in the paediatric cohort (n=27).

This shows that the most common etiology was CSOM, accounting for seven cases (25.9%). This was followed by foreign body ear, seen in six cases (22.2%). OME and otitis externa each contributed three cases (11.1%), while external ear mass and polyp were less frequent, with two cases each (7.4%). The remaining etiologies, pyogenic granuloma, thalassomycosisoma, lymphadenosis, and tumour, were all rare, each representing one case (3.7%).

**Figure 2:**
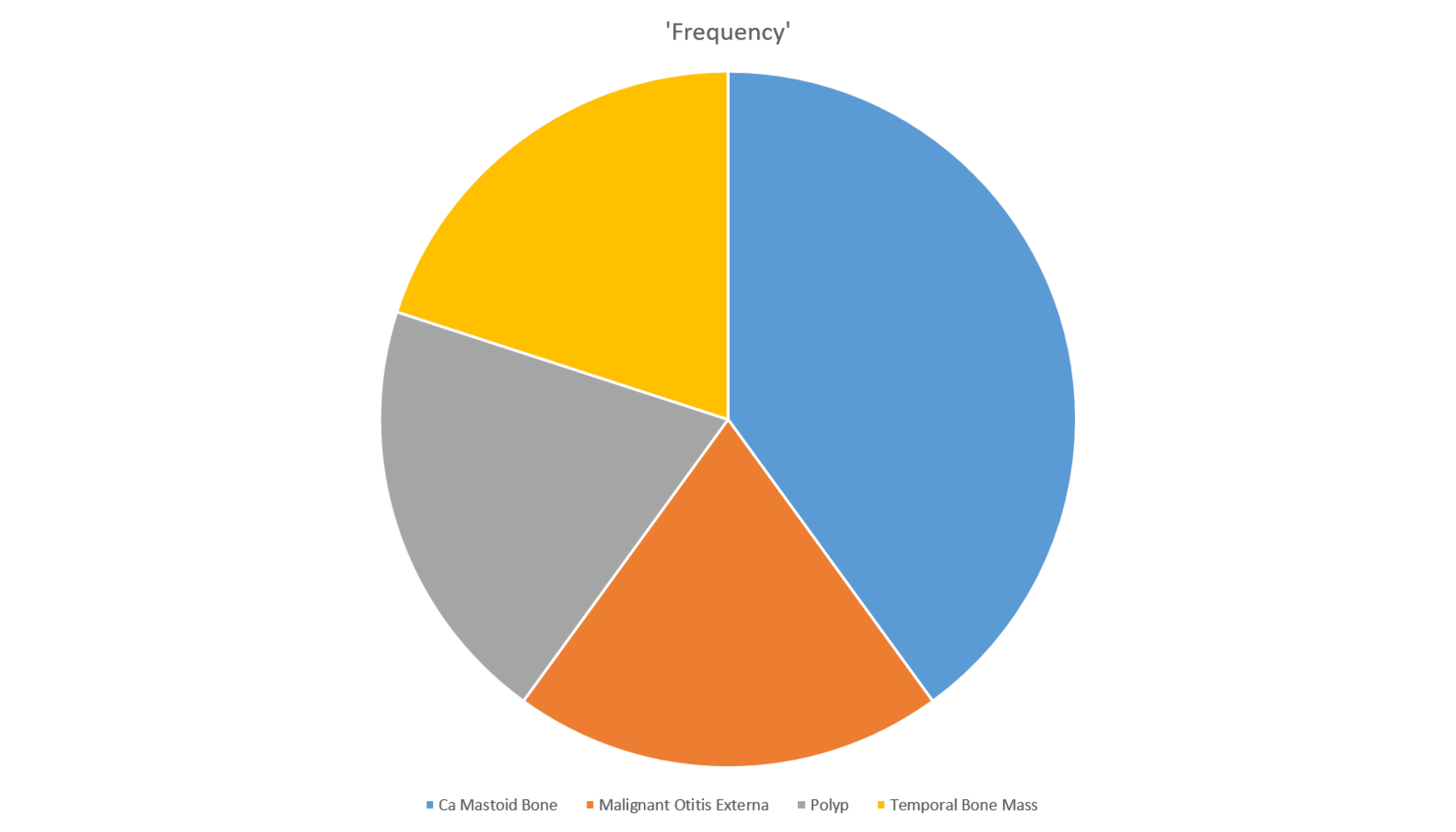
The distribution of etiologies in the geriatric population of the study

Though representing the smallest demographic subsection (n=5), the geriatric cohort (aged over 65) showed a distinct etiological pattern, as illustrated in Figure 2.

Unlike younger cohorts predominantly affected by infections like CSOM, 80% (n=4) of these elderly patients presented with space-occupying lesions or structural masses: specifically, one external auditory canal polyp, one temporal bone mass, and two mastoid bone carcinomas.

Severity and duration of hearing loss

The severity was determined by pure-tone audiometry averages and divided into mild, moderate, severe or profound. 

About 68.9% of patients presented with mild-to-moderate CHL whilst a smaller proportion at 13.2% presented with severe or profound hearing loss. Nineteen (17.9%) patients in the study did not have a pure-tone audiometry reading performed, and as such, the severity of their hearing loss could not be determined. These patients only had tuning fork testing done which identified them as having CHL. 

More than half of the patients (63.2%) reported symptoms persisting for longer than three months, indicating a high prevalence of chronic disease. The distribution of patients according to severity is described in Table 2.

**Table 2:** The distribution of patients according to hearing loss severity

Severity level	Frequency, n (%)
Mild	28 (26.4%)
Moderate	45 (42.5%)
Severe	11 (10.4%)
Profound	3 (2.8%)
Unknown (Tuning fork confirmation only)	19 (17.9%)
Total	106 (100.0%)

Associated symptoms

The most frequently reported symptom was ear discharge (66%), followed by tinnitus (58.9%) and ear pain (57.5%). Vertigo and ear itching were also commonly reported at 34% and 26.4%, respectively. Less frequent symptoms included headache, aural fullness, facial swelling, and facial nerve involvement. Table 3 describes the distribution of participants according to clinical symptoms.

**Table 3:** The distribution of participants according to clinical symptoms

Clinical symptoms	Frequency, n (%)
Discharge	70 (66.0%)
Tinnitus	62 (58.9%)
Ear Pain	61 (57.5%)
Vertigo	36 (34.0%)
Ear Itch	28 (26.4%)
Headache	10 (9.4%)
Facial Palsy	7 (6.6%)
Aural Fullness	5 (4.7%)
Aural Block	4 (3.8%)
Vomiting	2 (1.9%)
Dysphagia	1 (0.9%)
Facial Swelling	1 (0.9%)
Trismus	1 (0.9%)

Out of the 70 patients presenting with ear discharge, 36 (51.42%) had yellow discharge, six (8.56%) had green, five (7.14%) had white, four (5.71%) had bloody, two (2.86%) had clear, and two (2.86%) had cream-coloured discharge. The colour was not noted in 15 (21.45%) cases. The discharge was described as profound in 27 (38.6%) patients while being scanty in the other 43. Moreover, the smell was described as odourless in 41 patients while being offensive in 29.

Nasal involvement was seen in 10 patients, with four saying there was nasal congestion along with the hearing loss, four with a hypertrophied inferior turbinate on inspection, one claiming nasal discharge, and one with nasal obstruction.

Many of the etiologies stated previously can cause swelling in and around the ear, as seen in 21 patients. Out of these 21, there was swelling of the external auditory canal in 12 patients, postauricular swelling in five patients, preauricular swelling in two patients, and supraauricular temporal swelling seen in one patient each.

A previous history of hearing loss was noted in 56 (52.8%) patients. It was commonly seen in patients with a repeated history of otitis media and CSOM, as well as in four of the five cases of otosclerosis. A family history of similar symptoms was seen in seven (6.6%) patients.

## Discussion

This study evaluated the etiologies and clinical characteristics of CHL among patients presenting to Khartoum ENT Hospital and demonstrated that CSOM is the leading cause of CHL in this population. Nearly half of all cases were attributable to CSOM, highlighting the continued burden of preventable middle ear disease in low-resource settings. 

The heavy predominance of CSOM (47.2%) identified in our sample mirrors empirical findings from other developing contexts, such as regional studies across sub-Saharan Africa and South Asia, where overcrowding and delayed access reinforce infectious middle ear pathologies [4-7]. This distribution is consistent with previous institutional evaluations in Sudan by Khalifa et al. (2020), who identified infectious ear pathologies as dominant among paediatric cohorts aged 0-18 years [8], and Alrayah (2018), whose sample focused heavily on a young adult demographic [9]. Conversely, in high-income nations, non-infectious etiologies like mechanical otosclerosis (frequently accounting for 15-20% of adult CHL cases) or congenital ossicular anomalies are the primary clinical causes [1]. In our Sudanese cohort, otosclerosis was noted in only 4.7% of patients, while infectious markers dominated our clinical spectrum.

Similar to studies by Williamson (2011) and Orji (2013), most patients in this study presented with mild-to-moderate hearing loss, which aligns with previous reports indicating that CHL due to CSOM progresses gradually if untreated [6,10]. However, the high proportion of individuals presenting with chronic symptoms lasting over three months serves as a clear indicator of delayed healthcare-seeking behaviour. Because Khartoum ENT Hospital operates as a major tertiary referral centre, this prolonged timeline implies that patients face significant regional barriers to care, structural referral delays, or initial mismanagement at primary healthcare levels, forcing them to present only after conditions become chronic. Implementing early intervention protocols at the primary healthcare level could therefore bridge these gaps, potentially preventing progression to more severe hearing loss and advanced otological complications.

Ear discharge, tinnitus, and ear pain were the most frequently reported symptoms, reflecting classical clinical features of CSOM and other external and middle ear pathologies [11,12]. Vertigo was present in a significant proportion of the cohort, which may suggest labyrinthine involvement in more advanced or complicated cases of otitis media. This supports considering ear pathology in the differential diagnosis of vertigo, especially when hearing symptoms are present, as it may help pick up underlying or complicated middle ear disease earlier.

Foreign bodies of the ear and external auditory canal polyps were also notable causes of CHL, particularly among paediatric patients. In our cohort, which encompassed an age range of 0 to 17 years, these cases highlight a vulnerable demographic. This finding emphasises the need for parental education and early presentation when children develop ear-related symptoms. Otosclerosis, although less common, was observed mainly in young to middle-aged adults, consistent with its known epidemiological pattern [13]. 

Many studies carried out in Sudan highlight the low prevalence of tumours as a significant cause of hearing loss in the region. Within these prior investigations, such as the clinical profile compiled by Khalifa et al. (2020) at Khartoum Teaching Hospital [8], the epidemiological assessment by Alrayah (2018) in White Nile Province [9], and the etiological review by Taha et al. (2017) [14], only a single isolated case of a tumour causing hearing loss was recorded across their combined cohorts. In our study, however, benign and malignant tumours or masses made up 7.5% of all causes of hearing loss. Contrasting with these previous regional studies, our data demonstrate a substantially higher prevalence of tumours as a structural cause of CHL. This discrepancy is highly likely a function of selection bias, as our data is derived strictly from a specialized tertiary ENT referral hospital, which naturally attracts a dense concentration of advanced neoplastic and space-occupying lesions. In contrast, prior literature focused heavily on broader paediatric or primary care clinic populations [8,9]. 

A prior history of hearing impairment was identified in over half of the audited patients in this study, occurring in 52.8% of cases and predominantly among those presenting with CSOM. Inadequate treatment of acute otitis media is a recognised risk factor for progression to CSOM [15]. Within resource-limited healthcare settings, this clinical pattern is commonly exacerbated by systemic challenges in obtaining appropriate care or accessing essential medical supplies [4].

The over-65 population, although the smallest demographic subsection of this study (n=5), had the highest proportion of hearing loss due to a tumour or mass, with four out of five patients presenting with an aural polyp, temporal bone mass, or mastoid bone carcinoma. This creates a sharp epidemiological contrast within our data: while younger cohorts were heavily dominated by preventable infections like CSOM (47.2% overall), the geriatric population experienced a complete etiological shift toward neoplastic and structural changes. These findings support established literature indicating that as patients age, primary ear pathologies shift away from active infections toward tissue degenerations, cerumen impactions, and benign or malignant structural masses [16,17].

This study has several limitations. Its retrospective design relies on the accuracy and completeness of medical records, and the findings are based on a single tertiary center, which may limit generalisability. As well as this, as a correlational center-based study, it cannot prove direct causation. Nevertheless, the study provides valuable insight into the burden and characteristics of CHL in a major Sudanese referral hospital. 

## Conclusions

CSOM is the leading cause of conductive hearing loss among patients presenting to Khartoum ENT Hospital. Most patients had mild-to-moderate hearing loss with symptoms typical of chronic ear infection, including otorrhoea, otalgia, ear itch, tinnitus, and vertigo. As many cases are preventable or treatable when identified early, strengthening primary healthcare services, improving public awareness, and promoting early referral are essential to reducing the burden of CHL in Sudan.

To inform national hearing health policies, further multicentre and population‑based studies are recommended. These could include a multicentre prevalence study using standardised audiological assessments across several ENT centres to determine the national burden of CSOM‑related hearing loss, as well as a community‑based screening study within primary healthcare settings to identify early disease, assess risk factors, and evaluate barriers to timely care.

## Figures and Tables

**Figure 3:**
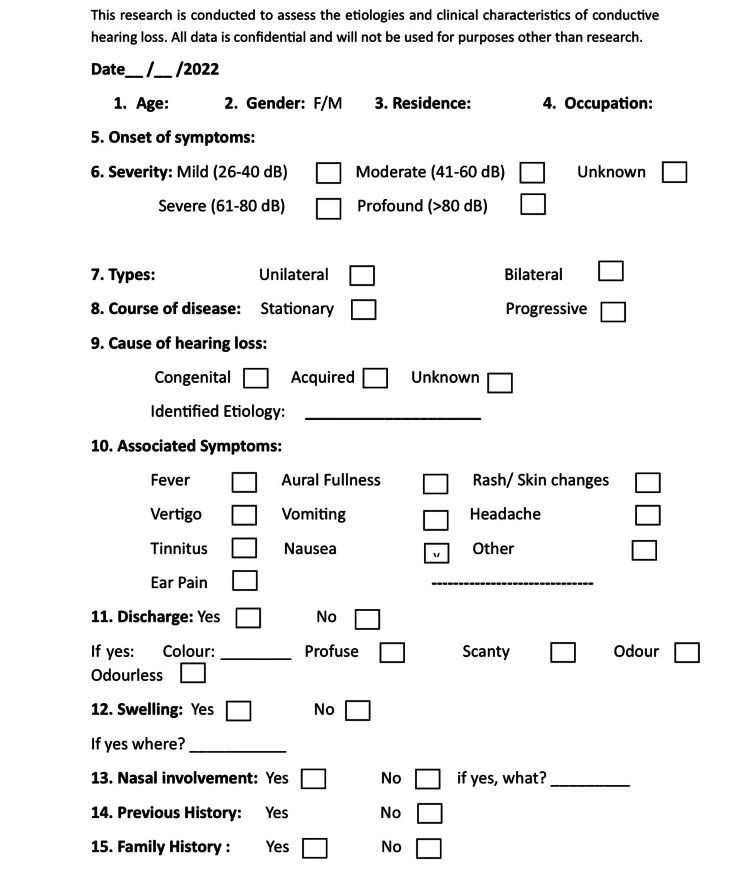
Research questionnaire
